# A Perspective on Emerging Therapeutic Interventions for COVID-19

**DOI:** 10.3389/fpubh.2020.00281

**Published:** 2020-07-03

**Authors:** Muhammad Torequl Islam, Md. Nasiruddin, Ishaq N. Khan, Siddhartha Kumar Mishra, Md. Kudrat-E-Zahan, Thoufiqul Alam Riaz, Eunus S. Ali, M. Safiur Rahman, Mohammad S. Mubarak, Miquel Martorell, William C. Cho, Daniela Calina, Anca Oana Docea, Javad Sharifi-Rad

**Affiliations:** ^1^Department of Pharmacy, Bangabandhu Sheikh Mujibur Rahman Science and Technology University, Gopalganj, Bangladesh; ^2^Department of Chemistry, Science Faculty, Bangabandhu Sheikh Mujibur Rahman Science and Technology University, Gopalganj, Bangladesh; ^3^Institute of Basic Medical Sciences, Khyber Medical University, Peshawar, Pakistan; ^4^Cancer Biology Laboratory, Department of Zoology, School of Biological Sciences, Dr. Harisingh Gour Central University, Sagar, India; ^5^Department of Chemistry, Rajshahi University, Rajshahi, Bangladesh; ^6^Department of Pharmacology, School of Medicine, Institute of New Drug Development, Jeonbuk National University, Jeonju, South Korea; ^7^Department of Biochemistry and Molecular Genetics, Northwestern University Feinberg School of Medicine, Chicago, IL, United States; ^8^Environmental and Atmospheric Chemistry Laboratory, Bangladesh Atomic Energy Commission, Dhaka, Bangladesh; ^9^Department of Chemistry, The University of Jordan, Amman, Jordan; ^10^Department of Nutrition and Dietetics, Faculty of Pharmacy, University of Concepcion, Concepcion, Chile; ^11^Centre for Healthy Living, University of Concepción, Concepción, Chile; ^12^Department of Clinical Oncology, Queen Elizabeth Hospital, Hong Kong, China; ^13^Department of Clinical Pharmacy, University of Medicine and Pharmacy of Craiova, Craiova, Romania; ^14^Department of Toxicology, University of Medicine and Pharmacy of Craiova, Craiova, Romania; ^15^Phytochemistry Research Center, Shahid Beheshti University of Medical Sciences, Tehran, Iran

**Keywords:** SARS-CoV-2, COVID-19 pandemic, public health, control, therapeutics

## Abstract

Coronaviruses are enveloped positive-sense RNA viruses with an unusual large RNA genome and a unique replication mechanism, which are characterized by club-like spikes that protrude from their surface. An outbreak of a novel coronavirus 2019 infection has posed significant threat to the health and economies in the whole world. This article reviewed the viral replication, pathogenicity, prevention and treatment strategies. With a lack of approved treatment options for this virus, alternative approaches to control the spread of disease is in urgent need. This article also covers some management strategies which may be applied to this virus outbreak. Ongoing clinical studies related to possible treatments for COVID-19, potential vaccines, and alternative medication such as natural compounds are also discussed.

## Introduction

The novel human coronavirus (SARS-CoV-2) seems emerged in December 2019 in Wuhan, China (1, 2). Later, on January 12, 2020, the World Health Organization (WHO) named it the 2019 novel Coronavirus (nCoV) and announced as a pandemic outbreak. SARS-CoV-2 is a member of β*-coronaviruses*. It is genetically related to the Severe Acute Respiratory Syndrome—Human coronavirus (SARS-CoV) and the Middle-East Respiratory Syndrome—Human coronavirus MERS-CoV (3–5).

The pandemic SARS-CoV-2 outbreak has caused infection in over 8,000,000 individuals and over 400,000 deaths in more than 200 countries across the world (6, 7). The number of infected cases is increasing at an alarming rate. The emergence of the SARS-COV-2 disease requires exploration and elucidation for a better understanding of its sources, production, transmission mechanism, prevention, management, and control (8).

Infection with this virus leads to respiratory damage, which can progress to pneumonia or damage to the whole body. The structure of the virus was identified in record time, and the mechanisms of infection were largely deciphered. These are the first steps to develop the most important infection control measures, in addition to prevention and hygiene (9).

This review highlights the latest knowledge on the sources, transmission, pathogenesis, prevention, and potential therapeutic control of SARS-COV-2/ COVID-19 disease. Literature review was performed to identify relevant articles published in English by April 1, 2020. The search terms used were: SARS-CoV-2, COVID-19, MERS-CoV, SARS-CoV, treatment, pharmacology, and efficacy. All types of articles were included. Clinical trials have been identified using the name COVID-19 disease on an index of studies into novel coronavirus pneumonia in the Chinese Clinical Trial Registry (10) and ClinicalTrials.gov.

## Coronavirus Disease 2019 (COVID-19): A Brief Overview

**Figure 1:**
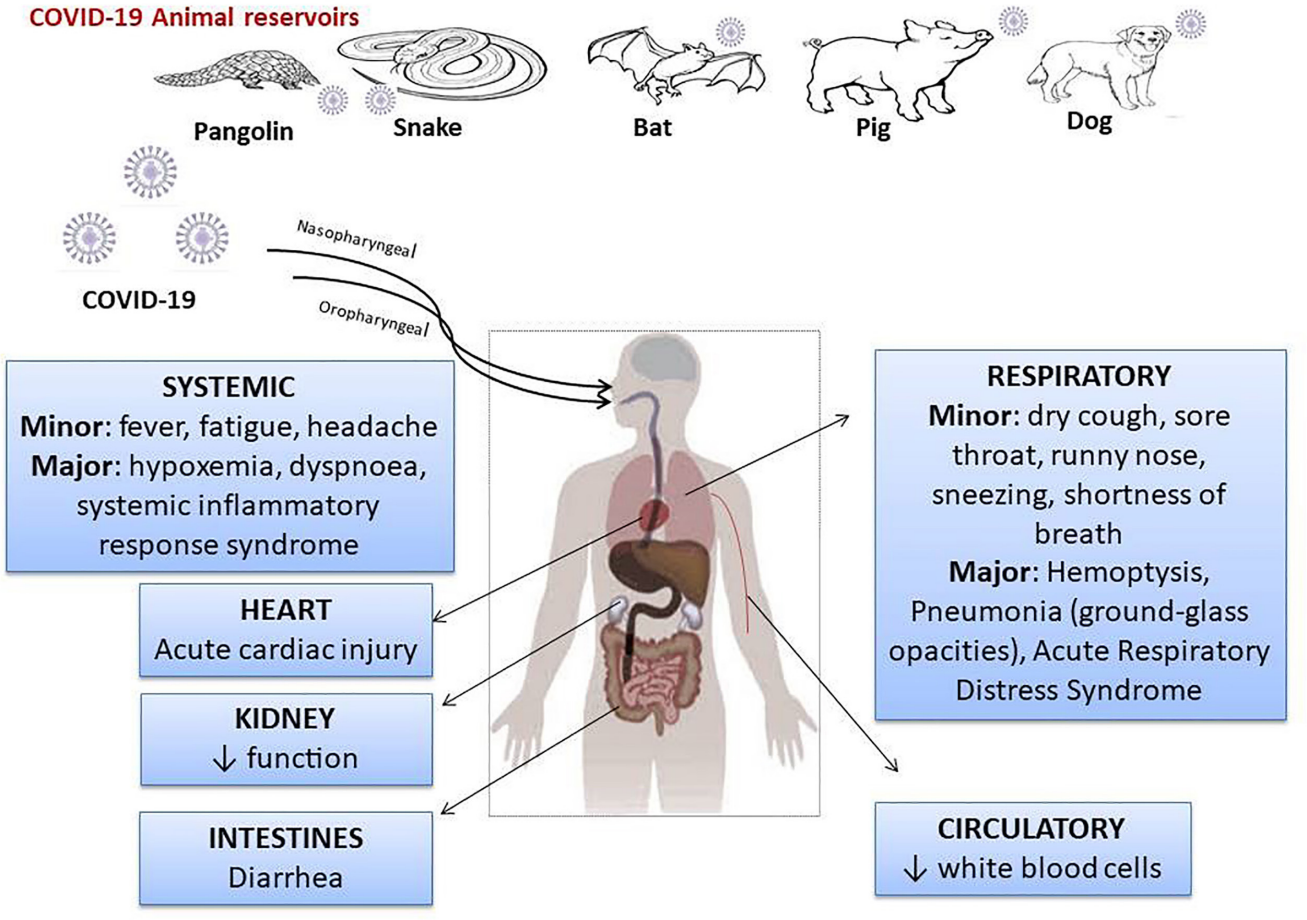
Probable sources of SARS-CoV-2 leading to symptoms, and prevention tips.

SARS-CoV-2 is a β-coronavirus with an envelope and genetic information in the form of an RNA. The non-structural proteins of SARS-CoV-2 are RNA polymerase, helicase, and proteases similar to 3-chemotrypsin and papain, and could be therapeutic targets. Surround the RNA molecule are the viral structural proteins, the most important of which is the S protein, which has the function of binding to the conversion enzyme of angiotensinogen II (ECA2), acting as a receptor in the case of SARS-CoV-2 and SARS viruses (11). In addition, protein S is modified by TMPRSS2 (transmembrane proteinase-serine 2), a modification that facilitates the entry of viral particles into the cell (12–14). The COVID-19 outbreak has been traced from live animals in “wet markets” in South China. In addition, reports indicated that SARS-CoV-2 may be transmitted to humans via pangolins (15, 16) or other wild animals [(17, 18); Figure 1].

The pathophysiological characteristics and the spreading mechanism of SARS-COV-2 remaining unclear. SARS-CoV-2 is mostly transmitted via inhalation (19) as the lung epithelial cells are the primary target of the virus.

**Figure 2:**
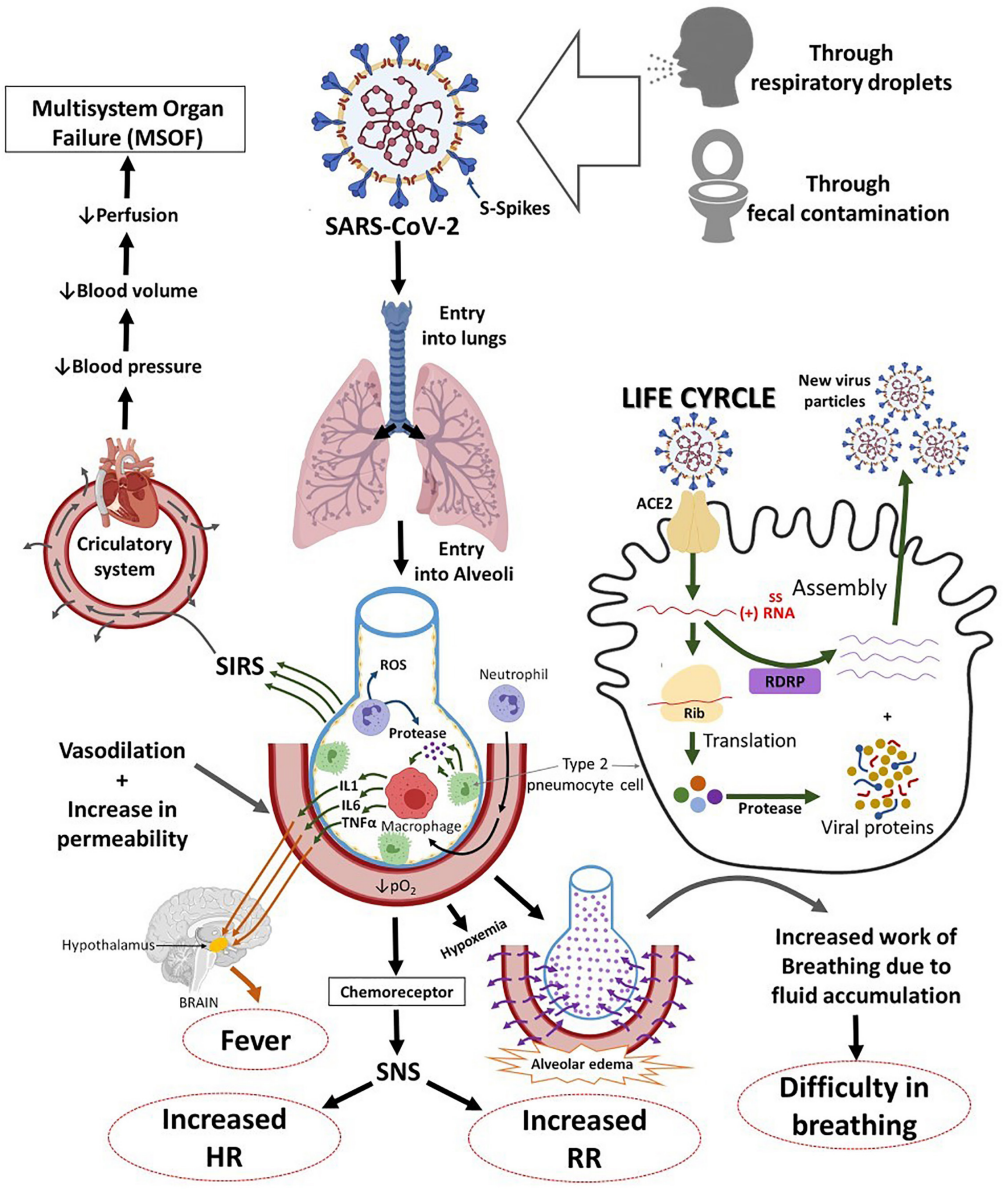
The systemic and respiratory disorders caused by SARS-COV-2 infection. The incubation period of SARS-COV-2 infection is ~5.2 days. There are general similarities in the symptoms between SARS-COV-2 and previous β-coronavirus. However, SARS-COV-2 shows some unique clinical features that include the targeting of the lower airway as evident by upper respiratory tract symptoms like rhinorrhea, sneezing, and sore throat. ACE2, angiotensin-converting enzyme 2; ssRNA virus, single-stranded RNA virus; RDRP, RNA-Dependent RNA Polymerase Gene; IL-1, interleukin 1; IL-6, interleukin-6; TNF-α, tumor necrosis alpha; PO2, partial pressure of oxygen; SNS, sympathetic nervous system; HR, heart rate; SIRS, systemic inflammatory response syndrome.

SARS-COV-2 may be manifested as an asymptomatic infection or mild to severe pneumonia (20). COVID-19 patients may experience abnormal respiratory findings, higher leukocyte numbers, and increased levels of plasma pro-inflammatory cytokines. It may also cause leucopenia, increased C-reactive protein, a high erythrocyte sedimentation rate, and high levels of pro-inflammatory cytokines [i.e., IL-2, IL-7, IL-10, GCSF, IP10, MCP1, MIP1α, and TNFα; (21)], a high serum aspartate aminotransferase (AST) or alanine aminotransferase (ALT), procalcitonin and ferritin levels (21, 22), decreased lymphocytes, elevated fibrinogen, neutrophil, lactic dehydrogenase, fibrinogen, and acute hypoxic respiratory failure (23). These findings suggest that immunopathology may also have a crucial role in the development of disease severity [(24, 25); Figure 2].

There is evidence that the SARS-CoV-2 virus could be transmitted by the fecal-oral route, not just by coughing and sneezing (26). Fecal viral RNA could be detected in some patients (27). Diarrhea is not the only gastrointestinal disorder described in COVID-19 diseases. Nausea and vomiting, discomfort or abdominal pain, and mild or moderate damage of the liver and pancreas (organs expressing ACE2) were also observed (28).

**Figure 3:**
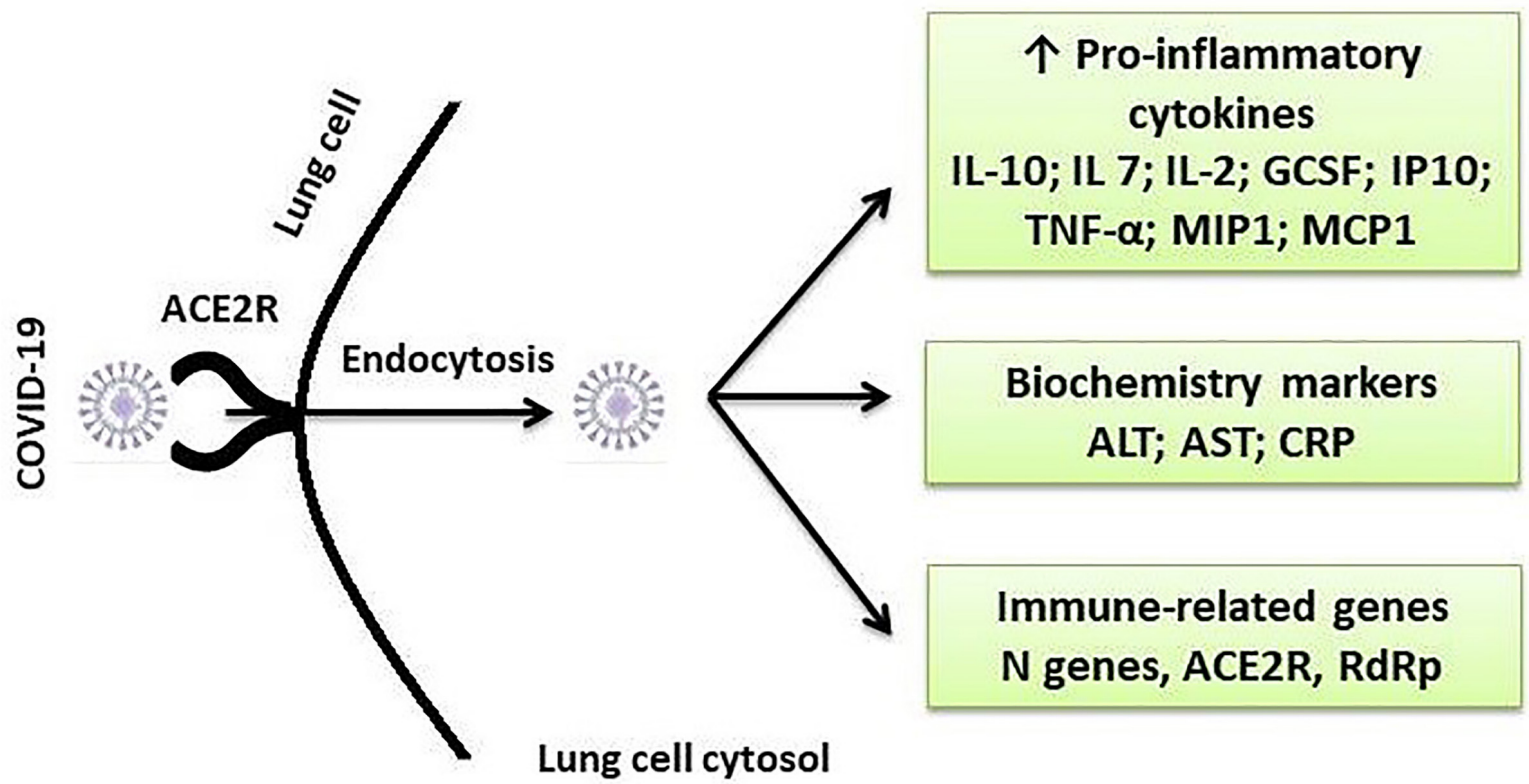
Some important diagnostic and therapeutic targets for SARS-CoV-2 infection. ACE2, angiotensin-converting enzyme 2; R, receptor; IL, interleukin; GCSF, granulocyte colony-stimulating factor; IP*-*10, interferon gamma-induced protein 10; TNF*-*α, tumor necrosis factor alpha; MIP1, Macrophage Inflammatory Protein 1; MCP-1, monocyte chemotactic protein-1; ALT, alanine transaminase; AST, aspartate transaminase; CRP, C-reactive protein.

In a COVID-19 laboratory diagnosis, serological tests use enzyme-linked immunosorbent assay (ELISA) or Western blotting that detects specific SARS-CoV-2 proteins, while molecular approaches are based on RT-PCR or Northern blot hybridization targeting specific SARS-CoV-2 genes (25). Direct immunofluorescence assay (IFA) is used to detect viral antigens present in the specimen, whereas the total lymphocyte count (TLC) and chest CT examination can also be used in SARS-CoV-2 infections (23). Some diagnostic features along with the treatment targets are depicted in Figure 3.

## Potential Therapeutic Targets and Treatments Using Drugs: Where we Stand Now

COVID-19 treatment should primarily aim for the rapid disappearance of symptoms, limiting interpersonal transmission and amelioration of severe forms at risk of death (29).

**Figure 4:**
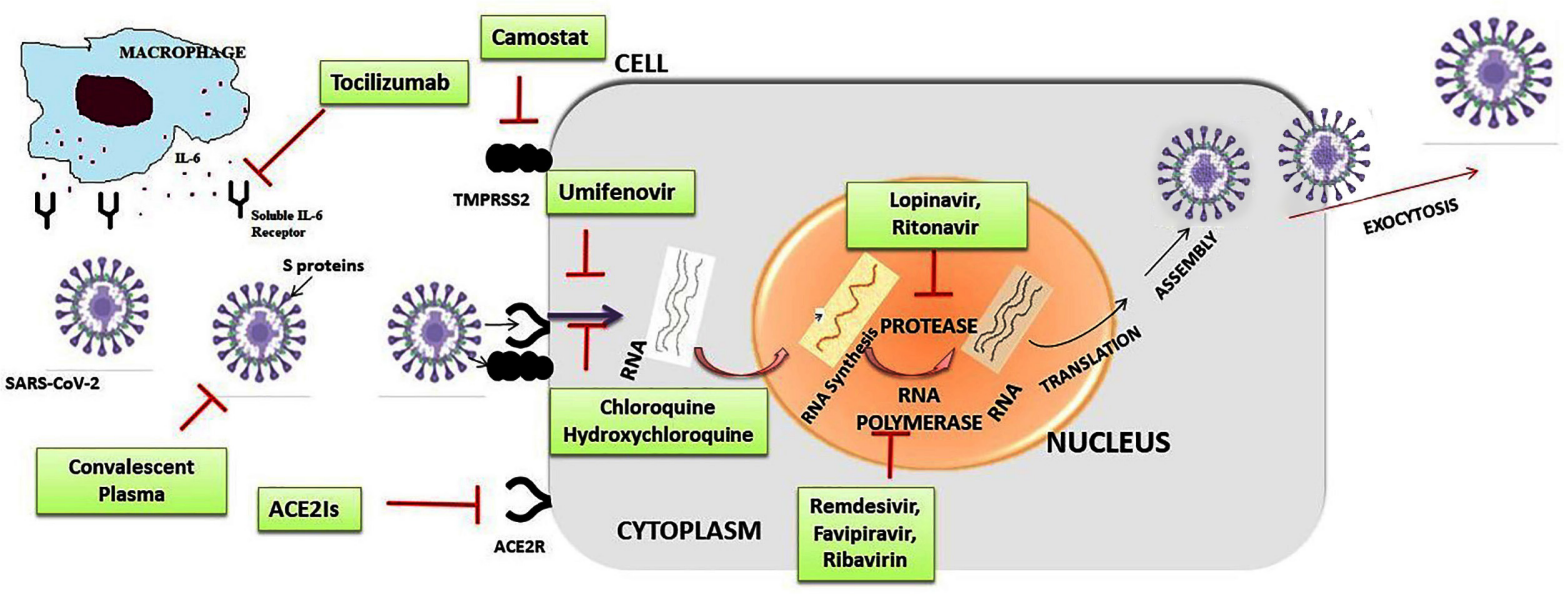
Summarized scheme with proposed acting targets of anti-SARS-CoV-2 in the replication cycle of the virus. Inhibitors of cell entry: inhibitors of angiotensin-converting enzyme 2 (ACE2Is) and antimalarial drugs: chloroquine, hydroxychloroquine; inhibitors of transmembrane protease/serine subfamily member 2 (TMPRSS2): camostate; Inhibitors of replication, membrane fusion, and assembly of SARS-CoV-2: remdesivir, lopinavir/ritonavir combination, umifenovir; humanized monoclonal antibody IgG1 anti-human receptor for interleukin-6 (IL-6): tocilizumab.

An effective treatment for SAR-CoV-2 can follow one of the following strategies (30): (i) Inhibition of functional enzymes or proteins, essential for the survival of the virus; (ii) Inhibition of viral structural proteins, preventing interaction with human cells or virion formation; (iii) Stimulating the immunity of the human host; and (iv) Inhibition of human proteins that act as receptors for the virus. For possible treatments for SARS-CoV-2 coronavirus infection, there are several molecules that could be effective against SARS-CoV-2 coronavirus in cell culture, animal, and early human trials [(31); Figure 4 and Table 1].

**Table 1:** The main drug classes used as potential treatments in COVID-19.

**Drug**	**Class**	**Rationale use**	**Clinical experience**	**Observations**
(Hydroxy)chloroquine	Antimalarial	Changes the pH of the cell membrane surface and thus inhibits the fusion of the virus with the cell membrane. Inhibits nucleic acid replication, glycosylation of viral proteins, assembly of the virus, and release of the virus from the infected cell.	*In vitro* activity against SARS-CoV-2, as well as some positive results in the treatment of patients with COVID-19 (32). A recent study showed faster virus clearance in patients with COVID-19 who received hydroxychloroquine (33). Initial dose: 600 mg (of chloroquine) followed by 300 mg (of chloroquine) 12 h later on day 1, then 300 mg (of chloroquine) twice daily on days 2–5.	It has been widely used in long-term treatments in rheumatology, without generating significant side effects.
Camostat	Inhibits TMPRSS2 protease	Inhibition of cell entry Prevents SARS-CoV-2 coronavirus infection	An *in vitro* study in a mouse model demonstrated the efficacy of camostat in protecting mice from death from a lethal SARS-CoV infection with a 60% survival rate (34). It is considered that doses of 600 mg (200 mg, three times) of camostat daily are expected to reduce SARS-CoV-2 infection; but human clinical trials are needed (35).	Mesylate camostat, approved in the treatment of inflammation of the pancreas in Japan.
Remdesivir	Antiviral for Ebola	Inhibits RNA-dependent RNA polymerase, prematurely blocking RNA transcription.	Broad antiviral spectrum; Efficacy against coronaviruses, both *in vitro* and *in vivo* studies; The safety profile has been demonstrated in Ebola studies; Superior efficacy of the Lopinavir/Ritonavir/IFNbeta combination in animal model studies. Adults and children weighing 40 kg or more: Loading dose of 200 mg by IV infusion on day 1, followed by 100 mg by IV infusion once daily on days 2–10 or followed by 100 mg by IV infusion once daily on days 2–5.	FDA (US) has authorized the use of remdesivir in severe infection with the new coronavirus SARS-CoV-2, through the Special Emergency Use Authorization (EUA).
Lopinavir /Ritonavir (LPV/RTV) combination	Antiviral for HIV	Lopinavir is a protease inhibitor used to treat HIV infection in combination with ritonavir to increase its availability.	Lopinavir has some degree of activity against coronaviruses *in vitro*, including SARS-CoV-2. The clinical data published so far have been inconsistent. Three observational studies failed to identify a reduction in the duration of virus excretion in patients treated with lopinavir/ritonavir compared to favipiravir or placebo, while during the Wuhan epidemic the use of lopinavir/ritonavir resulted in faster elimination of the virus. In the case of early administration, in the initial viral phase, in the first 10 days after the onset of symptoms (36). LPV/RTV (COVID-19): LPV 400 mg/RTV 100 mg orally twice daily for 10–14 days (37).	This drug remains as another alternative, in the absence of more effective drugs. An additional plus is related to the form of liquid administration—usable in orotracheal intubated patients and in newborns.
Umifenovir	Antiviral for influenza viruses	Blocking the penetration of the virus into cells (fusion inhibitor) and the immunomodulatory effect.	In patients with uncomplicated pneumonia in COVID-19, the combination of umifenovir (200 mg every 8 h) with lopinavir/ritonavir resulted in faster clearance of the virus at the nasopharyngeal level and a faster regression of lung imaging changes compared to patients receiving monotherapy with lopinavir/ritonavir (38). 200 mg orally 3 times daily for duration of 7–10 days or longer (39).	Reduced side effects
Favipiravir	Antiviral for flu and Ebola	RNA polymerase inhibitor	Used in China in patients of childbearing potential only if they had a negative pregnancy test and always associated with contraceptive medication during treatment and at least 7 days after stopping treatment; Doses: 1,600 mg every 12 h on the first day, then 600 mg every 12 h for 7–14 days. The drug cannot be administered to children and pregnant women (teratogenic risk)	Teratogenic effects
Tocilizumab	Immunomodulatory	IL-6 receptor antagonist	Has been used in a subset of patients with severe COVID-19 in whom there is excessive activation of inflammation (“cytokine storm”). The effectiveness of tocilizumab has been shown in small groups of patients; after administration of 1–2 doses, afebrility was obtained in all patients, as well as a decrease in oxygen requirements and partial correction of lymphopenia. The results obtained with tocilizumab combined with corticosteroids were favorable, following the administration of doses of 8 mg/kgc, repeated at 8–12 h, up to a maximum of 3 administrations (40).	Risks associated with the reactivation of tuberculosis, hepatic cytolysis, hypercholesterolemia.

### Inhibitors of Cell Entry

SARS-CoV-2 can only bind to ACE2 receptors (activated mainly in people with chronic diseases, while in healthy people ACE1 receptors are activated primarily) and TMPRSS2 protease to bind S (spike) receptors of the virus to ACE2 receptors. In the cell SARS-CoV-2 needs the TMPRSS2 protease, present in the human body, to enter into cells. So, this protease is a potential target for therapeutic interventions.

#### Inhibitors of Angiotensin-Converting Enzyme 2 (ACE2) and Antimalarial Drugs

Angiotensin converting enzyme (ACE2) is a possible therapeutic target due to its role as a viral ligand. ACE2 is commonly found in the cells of the epithelium and lung parenchyma, making it an accessible target for coronavirus, which is transmitted through the respiratory tract. In this regard, several molecules that could inhibit ACE2 have been identified, of which ruxolitinib is included in a clinical study together with mesenchymal stem cells (41).

Another way to act on the viral receptor is with the help of recombinant human ACE2 (rhACE2), a molecule studied in acute respiratory distress syndrome (ARDS), due to the high level of ACE2 in the lung. The study aims to study the effects of rhACE2 in patients with COVID-19, which could be beneficial both by lowering viremia (due to limited binding to the ECA2 receptor) and by protecting the lung from ARDS, which is one of the complications of COVID-19 with frequent fatal consequences (42).

Chloroquine [(N4-(7-Chloro-4-quinolinyl)-N1,N1-diethyl-1,4-pentanediamine)], a conventional drug for the treatment of malaria, has been applied in several studies against CoVs. In an early report, chloroquine was found to be effective in preventing the spread of SARS-CoV-2 *in vitro*. Chloroquine was assumed to elevate endosomal pH and to interfere with terminal glycosylation of the ACE2 receptor (43). This could negatively influence the virus-receptor binding to host cells by abrogating the infection, resulting in the inhibition of SARS-CoV-2 infection and spread (44). Emergence of the HCoV strains such as OC43 (HCoV-OC43) caused a 15–30% increase of mild upper respiratory tract infections. Research findings showed that chloroquine inhibits HCoV-OC43 replication in HRT-18 cells with LD_50_ effective concentration of 0.306 μM and an IC_50_ of 419 μM (45). In addition, chloroquine (15 mg/kg) could prevent the HCoV-OC43-induced death in newborn C57BL/6 mice with a high survival rate (98.6%) of the pups (46). This report advocated that chloroquine can be highly effective against HCoVs and it may be tested as a future drug against viral infection and spread.

The molecular mechanism of the action of chloroquine/hydroxychloroquine has been reported (47). First, drugs can change the pH of the surface of the cell membrane and thus inhibit the fusion of the virus with the cell membrane. Besides, they can also inhibit nucleic acid replication, glycosylation of viral proteins, virus assembly, transport of new virus particles, and release of the virus from the infected cell (48).

#### Inhibitors of Transmembrane Protease/Serine Subfamily Member 2 (TMPRSS2)

The therapeutic strategy, to inhibit human receptors of the virus, can be effective against the second protein in the penetration of the virus into cells—transmembrane proteinase TMPRSS2.

The camostat molecule, a synthetic serine protease inhibitor approved in Japan for pancreatic diseases (which also have proteinases as pathogenic elements), has demonstrated inhibitory effects on TMPRSS2 in cell culture studies and, implicitly, the inhibition of viral infection.

Another inhibitor, nafamostat, used as an anticoagulant and anti-pancreatitis agent and is approved for the treatment of cystic fibrosis. Nafamostat has mucolytic action and can prevent the deterioration of lung function (49).

### Inhibitors of Replication, Membrane Fusion, and Assembly of SARS-CoV-2

#### Remdesivir

According to the WHO, the most promising candidate for treatment of SARS-COV-2 is remdesivir (50, 51). Remdesivir is a nucleotide analog that acts against SARS-CoV-2 by inhibiting RNA polymerase and has the following advantages: a broad spectrum antiviral; efficacy against coronaviruses, both *in vitro* and *in vivo* studies; a safety profile demonstrated in Ebola studies; and a higher efficacy than the combination lopinavir/ritonavir/IFN β (Interferon beta) used in animal model studies (36, 45).

Recently, the US Food and Drug Administration (FDA) has authorized the use of remdesivir in infections with SARS-CoV-2, through the Special Emergency Use Authorization (EUA). This approval allows physicians to administer remdesivir to patients with a suspected or confirmed severe form of the infection (those who have blood oxygen saturation SpO2 ≤ 94%, require oxygen therapy, mechanical ventilation, or extracorporeal membrane-ECO oxygenation/ECMO), even outside of clinical trials. However, EUA is not a complete approval, as further studies are needed to confirm the effectiveness of this treatment.

Urgent approval follows the publication of encouraging results from two studies involving remdesivir:

The ACTT study, organized by the US National Institute of Allergic and Infectious Diseases (NIAID) was a phase III, randomized, placebo-controlled trial with 1,063 patients included. Results of the study found that patients treated with remdesivir showed a clinical improvement after a 31% shorter period. The study group had a median recovery time of 11 days, compared to 15 days in the control group. The study group had a mortality of 8% compared to 11.6% in the control group (52).

The SIMPLE study, organized by Gilead (the company that produces remdesivir) was a phase III trial without a control group in which patients received a remdesivir treatment for 5 or 10 days. Results showed that clinical improvement was similar in the two groups. Half of the patients showed an improvement in the disease in the first 10 days, in the case of 5-day treatment, and in the first 11 days with the 10-day treatment; after 14 days, 60% of patients receiving remdesivir for 5 days were discharged, and 52.3% of those receiving 10 days were discharged (53).

#### Lopinavir /Ritonavir Combination

The combination of lopinavir/ritonavir protease inhibitors (marketed as Kaletra for the treatment of HIV infection), with or without IFNβ, is another viable candidate in the fight against SARS-CoV-2 (54). It is already included in the MIRACLE study against the MERS virus, and the first study in China against SARS-CoV-2 has been started. In addition, a team of experts from Wuhan (the city where the infection began) developed a best practice guide, following the management of a large number of patients. In addition to supportive treatment, the guide includes the use of lopinavir + ritonavir + IFN, which is, however, supported by only a low level of scientific certainty (the recommendation is based on use in SARS and MERS infections, related but not identical, to SARS-CoV-2) (55).

Lopinavir/ritonavir works by inhibiting the 3-chemotrypsin-like protease of SARS, MERS, and SARS-Cov-2. Observational studies (in SARS-CoV-2 and SARS) support the initiation of therapy in the first 7–10 days, otherwise the clinical benefits are not found in patients (36).

In addition to the combination with IFN, the two protease inhibitors are also administered in studies with ribavirin (a guanosine analog and RNA synthesis inhibitor that could inhibit papain-like proteinase in COVID-19), emtricitabine/tenofovir (other approved therapies against HIV, which inhibits the enzyme reverse transcriptase), or with umifenovir (inhibitor of viral fusion of human cell membranes, which inhibits the interaction between protein S and the ECA2 receptor).

#### Umifenovir

Umifenovir (Arbidol) has an effect against influenza viruses and the mechanism of antiviral action is based on blocking the penetration of the virus into cells (fusion inhibitor) and the immunomodulatory effect (56). One of its advantages is its reduced side effects. Umifenovir has been tested in combination with other antivirals in patients with uncomplicated pneumonia with COVID-19 (57).

The combination of umifenovir with lopinavir/ritonavir was found to result in faster clearance of the nasopharyngeal virus and a faster regression of lung imaging compared to patients receiving lopinavir/ritonavir monotherapy (58).

#### Favipiravir

Favipiravir is an RNA polymerase inhibitor that has been used for influenza and Ebola infection.

It was originally produced in Japan and used more frequently in China; due to its teratogenic effect, in Japan its use is only allowed during the evolution of emerging epidemics or infections. In SARS-Cov-2 infection, favipiravir was more effective in viral eradication and regression of lung imaging than both lopinavir/ritonavir and of umifenovir. However, it can only be given to women who are not pregnant (59).

### Neuraminidase Inhibitors

Oseltamivir, peramivir, or zanamivir are not justified for the treatment of COVID-19, because this virus has no neuraminidase; the combination of anti-flu medication is recommended for patients with COVID-19 until an exclusion diagnosis of influenza by gene amplification test is carried out or for as long as necessary for treatment of a concomitant infection with an influenza virus (60).

### Tocilizumab

Tocilizumab is a humanized monoclonal antibody IgG1 anti-human receptor for interleukin-6 (IL-6), obtained by recombinant DNA technology in Chinese hamster ovary (CHO) cells. The drug tocilizumab (trade name Actemra) is therapeutically indicated for patients with rheumatoid arthritis, but Chinese authorities have stated that it can be prescribed for patients with coronavirus infection who have severe lung damage and high levels of interleukin 6 protein (IL-6), which indicates inflammation and immune disorders (61).

This immunomodulator may be used in a subgroup of patients with severe forms of COVID-19 in that there is excessive activation of inflammation (“cytokine storm”). Identifying patients who would benefit from taking tocilizumab can be based on parameters such as growth ferritin levels, decreased platelet count, and increased ESR and C-reactive protein (61).

### Convalescent Plasma: A Potential Treatment for COVID-19

Passive immunotherapy dates back to the 1890s. It was the only way to treat many infectious diseases before the advent of antimicrobial therapy in the 1940s. The convalescent plasma was also used during the 2013 African Ebola epidemic (62).

Experience from previous coronavirus situations, such as SARS-Cov-1, shows that convalescent plasma contains neutralizing antibodies to the virus.

In the case of SARS-CoV2, the main mechanisms of action of passive antibody therapy are antiviral and immunomodulatory (63).

Antibodies could work in several ways:

- Viral neutralization: the antibody attaches to the virus and kills it.
- Antibody-dependent cellular cytotoxicity: the antibody stimulates a specialized immune cell to target the virus and attack its membrane, ultimately causing the virus to disintegrate.
- Antibody-dependent cellular phagocytosis.

Passive immunotherapy includes the administration of antibodies against pathogens in susceptible or infected indices for the purpose of preventing or treating the disease due to the pathogen. Currently the only antibody available for immediate use in the potential treatment of COVID-19 is found in plasma taken from cured patients (64).

In contrast, active vaccination induces an immune response that takes time to develop, with variable responses depending on the patient. Thus, immunocompromised patients fail to obtain an adequate immune response. Therefore, passive administration of antibodies is the only way to produce immediate immunity for susceptible individuals and for immunocompromised patients (65).

Several countries, such as the United States (66) and the United Kingdom (67), have announced initiatives to treat patients hospitalized with COVID-19. The priority sampling procedure is plasmapheresis, but whole blood sampling can also be used, with subsequent separation of the plasma from it. Plasma is tested for HIV, HBV, HCV, and anti-HLA antibodies (in some donors), and administered to patients with severe forms of COVID-19 hospitalized in ATI, with rapid disease progression of> 50% in 24–48 h (with lung damage, mechanically ventilated or requiring mechanical ventilation soon, due to dyspnea, tachypnea, and low oxygen saturation).

In addition to the direct use of plasma from recovered patients, it can be used to perform a treatment consisting of polyclonal hyperimmune immunoglobulin.

## Ongoing Clinical Studies Related to Possible Treatments for COVID-19

MIRACLE is the first trial to have begun in China against SARS-CoV-2. The combination of lopinavir/ritonavir protease inhibitors (marketed under the name Kaletra for the treatment of HIV infection), with or without IFNβ, is a viable candidate in the fight against SARS-CoV-2. A team of experts from Wuhan (the city where the infection occurred) developed a good practice guide, following the management of a large number of patients (68). In addition to supportive treatment, the guide includes the use of lopinavir + ritonavir + IFN, which is supported, however, by only a low level of scientific certainty (the recommendation is based on the use of SARS and MERS infections, related to, but not identical to, SARS-CoV-2-2). Lopinavir/ritonavir acts by inhibiting the protease similar to 3-chemotrypsin, from the structure of SARS, MERS, and SARS-Cov-2. In addition to the combination with IFN, the two protease inhibitors are administered in studies and in combination with ribavirin (guanosine analog and RNA synthesis inhibitor, which within SARS-COV-2 could inhibit papain-like proteinase), emtricitabine/tenofovir (other approved therapies against HIV, which inhibit the enzyme reverse transcriptase), or with umifenovir (inhibitor of viral fusion by human cell membranes).

SOLIDARITY trial is a study conducted by WHO and the first patients with SARS-COV-2 were already included (69). The purpose of the study is to identify the most effective antiviral agent against SARS-CoV-2, between lopinavir/ritonavir (with or without interferon beta), and remdesivir and chloroquine (or hydroxychloroquine). Spain and Norway are the countries that have administered the treatment, out of the 45 who have joined this project, so far. The Norwegian component of the SOLIDARITY study is registered on the official Clinical Trials platform and provides more details on the progress of the study; the first patients included in Norway are part of the proposed cohort of 700 patients, who will be randomly assigned to three study subgroups: remdesivir, hydroxychloroquine, and standard (supportive) treatment. According to WHO indications, the objectives pursued are mortality, length of hospitalization, and the proportion of patients who require intensive medical support in the intensive care units. In addition, Norwegian doctors will also track the rate at which the virus is eliminated from the body (viral clearance from blood and respiratory samples) and biological markers of its impact on the body (inflammation, endothelial, and platelet activation) (69).

Trial of Treatments for COVID*-*19 in Hospitalized Adults (DisCoVeRy) study will be conducted in Europe by the National Institutes of Health^1^ and Medical Research in France (Inserm), and it will include the same therapeutic molecules and a total of 3,200 patients from Belgium, Germany, Luxembourg, the Netherlands, Spain, Sweden, and the United Kingdom. Several medical centers will start collecting data as new cases emerge, and the first data analysis will be performed after 15 days of treatment (70).

COVACTA is a new study that aims to identify a potential treatment against SARS-COV-2. The drug that will be investigated is tocilizumab, a monoclonal antibody that inhibits interleukin 6 receptor and is used in rheumatology. The study will be a randomized, double-blind, placebo-controlled, phase III trial that will track the benefits of tocilizumab added to the standard of care (ventilator support). The study will include 330 patients worldwide and will follow their clinical status, along with the proportion of patients requiring intensive care, mechanical ventilation, or developing severe forms of SARS-COV-2, which evolves with death (71). This study is the result of case studies, where it has been observed that tocilizumab improves the clinical status in severe cases of SARS-COV-2, with advanced pulmonary inflammation. This use is based on the approval of tocilizumab as a treatment in cytokine release syndrome (CRS), an inflammatory manifestation throughout the body, which may be an adverse reaction to CAR-T cell immunotherapy. In the case of SARS-COV-2, the penetration of SARS-CoV-2 into the body stimulates an immune response, with cytokine release (including IL-6), which stimulates fever, inflammation, and pulmonary fibrosis. Studies of SARS-COV-2 cases have shown that elevated levels of IL-6 in patients' serum are statistically significantly correlated with the severe evolution of the infection (71).

To test the impact of camostat mesilate on COVID-19 Infection (CamoCO-19), an Investigator-initiated Randomized, Placebo-controlled, Phase IIa Trial is underway and its results are expected to be announced in December this year (72).

The efficacy of nafamostat in patients with Covid-19 (RACONA study), is the subject of another ongoing clinical trial. The aim of the RACONA study is to test the hypothesis that nafamostat is useful in the treatment of COVID-19 lung damage. This hypothesis is justified by the fact that COVID-19 involves the activation of the coagulation cascade, pulmonary embolism, and bacterial superinfections (73).

Chloroquine phosphate displayed apparent efficacy and acceptable safety against SARS-COV-2 in multicenter clinical trials conducted in China (74). The use of this drug appears in the Guidelines for the Prevention, Diagnosis, and Treatment of Pneumonia Caused by SARS-CoV-2 issued by the National Health Commission of the People's Republic of China. Another recent study suggested chloroquine phosphate tablet (500 mg twice per day for 10 days) against SARS-COV-2 (74). Although chloroquine has long been used to treat malaria and amebiasis, *Plasmodium falciparum* has developed widespread resistance to it (75). Furthermore, an overdose of chloroquine was known to cause acute poisoning and death (76) which limits its utilization in clinical practices.

Hydroxychloroquine is a derivative of chloroquine, and significantly less (~40%) toxic (77). Recently, hydroxychloroquine was found to efficiently inhibit the SARS-CoV-2 infection *in vitro* through an anti-inflammatory mechanism (48). In a most recent report, hydroxychloroquine's role on respiratory viral loads was evaluated using SARS-COV-2 patients from France (78). Hydroxychloroquine (600 mg daily) was administered to patients and their viral load in nasopharyngeal swabs was tested daily. After 6 days of treatment, 20 cases showed a significant reduction of the viral load. Interestingly, the addition of azithromycin to hydroxychloroquine significantly eliminated the virus as compared to a single therapy (79). This clinical survey demonstrated that hydroxychloroquine treatment is significantly associated with viral load reduction in SARS-CoV-2 infected patients, with better results obtained by the addition of azithromycin (78). This might be a milestone preventive option in limiting the infection and spread of SARS-CoV-2.

In the case of umifenovir (an active antiviral against influenza, approved only in China and Russia), its use in COVID-19 is promising; according to ongoing Chinese studies, it could lead to a lower mortality rate. A randomized study compared umifenovir with another promising antiviral, favipiravir, which resulted in a higher cure rate after 7 days of treatment in moderate cases of COVID-19 (71.4% in the favipiravir group, compared to 55.9% in the umifenovir group) (80).

Other therapeutic options, with lower chances of success—according to the WHO—are monoclonal or polyclonal antibodies, or serum collected from patients with SARS-COV-2, which contains antibodies against infection (25). However, a study involving plasma harvested from patients cured of SARS-COV-2 and its administration in severe cases of pulmonary disease has been initiated—in which a few cases of clinical remission have been described (81).

WHO has also identified a number of molecules into which studies are discouraged—ribavirin and immunosuppressants, such as corticosteroids (which may be useful in severe lung injury), and in the case of chloroquine there is insufficient evidence to substantiate the studies. However, studies including these drugs have been started, based on preclinical evidence of action against SARS-CoV-2 virus (82, 83).

Last but not the least, a number of non-specific treatments can bring about clinical improvements, such as statins, heparin, and vitamin C. In addition to the molecules described by WHO, recent studies suggest other possible treatments for SARS-CoV-2. Mesenchymal stem cells are the subject of several phase I and II studies, carried out within the SARS-CoV-2 infection. The therapeutic hypothesis is that the immunomodulatory and regenerative properties of stem cells can inhibit the inflammatory component of SARS-COV-2 lung disease (which can lead to fatal disease progression) (84).

Elements of traditional Chinese medicine (TCM), such as herbal preparations or acupuncture, are included in the Wuhan Good Practice Guide and are the subject of several clinical studies initiated in SARS-COV-2 (85).

## Vaccines Against COVID-19: New Hope for Public Health

Worldwide, there is increasing attention of identifying vaccines that could be the salvage solution against COVID-19. In March 2020, many pharmaceutical companies started developing vaccines worldwide. One month later, in April 2020, the first vaccine in clinical trial in humans began.

A vaccine is a biological preparation used to produce or enhance immunity against a particular disease, such as COVID-19. Inoculation of dead or weakened microorganisms of the virus that causes the disease (or fragments, products, derivatives) stimulates the production of antibodies (86). When the immune system encounters the microorganism that causes the disease, then it itself prevents the disease from reacting quickly and efficiently (87).

The human immune system is a system of biological structures and processes that protect us against disease, by recognizing germs that enter the body as foreign invaders. When antigens invade the human body, the immune system responds by producing protein substances called antibodies and very specific cells that can fight off invading germs (88).

Immunity is the successful defense of the body against a pathogen. When the body has produced a sufficient number of antibodies to fight the disease, this immunity results, providing protection against the disease for many months, years, or even life. If a person subsequently comes into contact with the same pathogen, the immune system will be able to rapidly produce the same type of antibody that prevents the disease from developing or decreases its severity and allows it to remove the pathogen from the body. By “immunological memory,” it is estimated that the immune system can remember or effectively recognize and fight hundreds of thousands, possibly millions, of different foreign organisms (89).

Vaccination involves the introduction of a limited amount of disease-specific antigens into the human body, which stimulates the immune system sufficiently to produce the required amount of antibodies but without causing the disease (90).

Vaccine development is a complex and time-consuming process that differs from conventional drug development. Indeed, vaccines are intended for use in healthy people as a preventive measure, while conventional medicines are geared toward treating a disease. Clinical studies to demonstrate the efficacy of a vaccine focus on demonstrating that it can prevent the disease, which implies the need for a greater number of subjects than in the case of traditional drug studies. Before a vaccine is approved and brought to market, it goes through a long and rigorous research process, followed by many years of testing to meet stringent regulatory requirements. Thus, clinical trials for vaccines are carried out in three research phases. Phase I involves a small number of volunteers (20–50 people) and aims to evaluate safety, determine dosage, and identify potential adverse reactions. In phase II of the clinical studies about 100–300 volunteers are involved, the purpose being to analyze in more detail the safety and immunogenicity, the necessary dosage, and to identify the administration schedule. Phase III studies include 3,000–50,000 volunteers, being the last phase to evaluate the safety and efficacy of the large-scale vaccine and to analyze the concomitant administration with other vaccines and treatments. After the testing phase, the vaccines must be approved by the regulatory agencies, in our case by the European Medicines Agency, and only then can they reach the doctors and the population (91).

The latest data on obtaining a vaccine against SARS-CoV-2 come from researchers at the University of Oxford who have announced that they have started enrolling healthy participants in a clinical trial to test a candidate vaccine for COVID-19, called ChAdOx1 nCoV-19.

Initially developed to prevent MERS, this potential vaccine is based on an adenovirus vaccine vector and COVID-19 spike protein. Currently, it is being manufactured in the University of Oxford's Clinical Biomanufacturing unit and will be ready in a few weeks, according to trialsitenews.com.

Developed at the University of Oxford, ChAdOx1 nCoV-19 is a safe version of an adenovirus. It has been modified so that it cannot be reproduced in the human body and the genetic code that transmits instructions for the production of Coronavirus Spike protein has been added, allowing the adenovirus to produce this protein after vaccination. The result is the formation of antibodies against Spike protein, known to be on the surface of SARS-CoV-2 (92).

The clinical trial will enroll up to 510 participants and will be led by the Jenner Institute and the Vaccinology Group of the University of Oxford. The study started in March 2020 and is scheduled to be completed in May 2021. It will be conducted in the UK, on healthy adult volunteers aged 18–55. The vaccine will be administered intramuscularly.

Official reports show that university researchers are tracking results in additional preclinical tests to assess safety, while pledging to invest in the production of a large number of units prior to clinical trial. The Oxford team of researchers has significant experience in contributing to addressing public health emergencies, having been active during the 2014 Ebola outbreak (93, 94).

China has already announced that it has a vaccine clinical trials. The potential candidate is realized by the Military Academy of Medical Sciences of China and Cansino Biologics and is based on a technological platform developed by Cansino, related to viral adenoviruses. It is the same platform where a vaccine for Ebola was successful in 2017 (95).

Ad5-nCoV is a novel vaccine, developed by genetic engineering with the replication of type 5 adenovirus as a vector of immunity against the protein of the new CoV (96).

Other candidate vaccines are currently in the preclinical study phase. Johnson & Johnson initiated research efforts for the various potential candidate vaccines in January 2020, as soon as the new coronavirus sequence (COVID-19) became available. Janssen research teams, in collaboration with Beth Israel Deaconess Medical Center, part of Harvard Medical School, have developed and tested several vaccine candidates using Adansac® Janssen technology (97). Following this approach, Johnson & Johnson has identified a prime candidate for the COVID-19 vaccine (with two spare variants), leading to the first stages of production. With an accelerated track record, the company intends to launch the Phase 1 trial in September 2020, and clinical safety and efficacy data are expected to be available by the end of the year. This process would allow vaccine availability for emergency use in early 2021. For comparison, the usual process of developing a vaccine involves a number of different research stages, over a period of 5–7 years, before a candidate could be considered for approval (97).

Kentucky Bioprocessing (KBP) is developing a possible vaccine for COVID-19, currently in the pre-clinical testing phase (98). The developing vaccine uses a BAT technology for rapid growth of the tobacco plant, with several advantages over the conventional vaccine production technology: (i) It is potentially safer, given that tobacco plants cannot harbor pathogens that lead to human disease; (ii) It is faster because the vaccine elements accumulate in tobacco plants much faster−6 weeks in tobacco plants compared to several months when conventional methods are used; (iii) the vaccine formula developed by KBP remains stable at room temperature, as opposed to conventional vaccines, which often require refrigeration; and (iv) it has the potential to deliver an effective single-dose immune response.

KBP recently cloned a portion of the genetic sequence of COVID-19, which led to the development of a potential antigen—a substance that induces an immune response to the body and particularly stimulates antibody production. This antigen was then inserted into tobacco plants for reproduction. After the plants were harvested, the antigen was purified and is currently undergoing pre-clinical testing (99). The pharmaceutical industry and health authorities argue that there are significant efforts to diagnose, treat, and prevent infections with the new coronavirus.

## Alternative Medication—Natural Compounds To Control SARS-CoV-2: *IN VITRO* Studies

Along with therapeutic drug development and vaccine trials, it has become necessary to search for possible alternative and integrated medicinal systems involving natural products to treat SARS-CoV. Some natural products with immunostimulatory and antiviral action are recommended in respiratory viruses and viral infections with various locations (100, 101). They support immunity and strengthen the body by protecting it from viruses. TCM and other traditional and complementary medicine systems have a range of herbal preparations that could be assessed in combination with synthetic drugs for preventing and treating SARS (102). These could serve as a cure and could prevent infection and viral replication. Some TCM and other herbal preparations could resolve toxic responses, eliminate pathological dampness, and improve lung function and blood circulation (102–105). In addition, some TCM, such as Snow Lotus (*Saussurea involucrata* Matsum. & Koidz.), may enhance immunity and be beneficial for CoV infection treatment (106). However, further evidence is needed.

Several traditional herbal medicines have been reported for their plausible antiviral activities against SARS-CoV-2 (103, 107–109). Glycyrrhizin and it derivatives from liquorice roots (*Glycyrrhiza glabra* L.) were found to exhibit antiviral activities against SARS-CoV-2 (110–112). In combination with herbal preparations, indomethacin, a non-steroidal anti-inflammatory drug, showed potent antiviral activity against SARS-CoV-2 (113). These formulations might stop the replication of SARS-CoV-2 through inhibition of one or more viral proteins including SARS-CoV-23CL protease. This protease is an important factor that regulates the proteolytic processing of replicase polypeptides into functional proteins, and plays a key role in viral replication (114, 115). Thus, SARS-CoV-23CL protease can be a suitable target for drug candidates against SARS-CoV.

Along this line, an in-depth study evaluated more than 200 herbal extracts from TCM for antiviral potentials against SARS-CoV-2 on Vero E6 using a cell-based assay cytopathogenic effect (116). Among these, six herbal extracts of plants and plant parts *Gentiana scabra* Bunge (the dried rhizome), *Dioscorea polystachya* Turcz. (the tuber), *Senna tora* (L.) Roxb. (the dried seed), *Taxillus chinensis* (DC.) Danser (the dried stem with leaf), and *Cibotium barometz* (L.) J.Sm. (the dried rhizome) were found to be potent inhibitors of SARS-CoV-2 at concentrations ranging between 25 and 200 μg/mL. Similarly, two extracts of *C. barometz* also showed notable inhibition of SARS-CoV-23CL protease activity with IC_50_ values of 39 and 44 μg/mL, respectively (116). These herbal extracts inhibited replication and 3CL protease activity of SARS-CoV, thus suggesting that such specific herbal extracts may be potentially utilized as drug targets for future antiviral drug development against SARS-CoV. *G. scabra* was also reported for its hepatoprotective effect because of the triterpenoids of secoiridoid and its glycosides (117, 118) which adds to liver-protection during hepatic failure due to viral proliferation.

On the basis of these observations, other specific triterpenoids have also been reported to inhibit SARS-CoV-2 replication, especially secoiridoid and its glycosides from *G. scabra* extract (116). Two other specific polysaccharide-containing fractions from *D. polystachya* tuber extracts were reported to remarkably increase the GM-CSF promoter activity in improving the regeneration of bone marrow cells, and exerted anti-inflammatory effects through the inhibition of NF-κB-mediated iNOS and COX-2 expressions (119–122). The inflammatory pathways involving COX-2 may correlate with antiviral activity against SARS-CoV-2 and other antiviral activities (113, 123). Similarly, emodin, a trihydroxyanthraquinone obtained from rhubarb, buckthorn, and Japanese knotweed, exhibited antiviral activity against SARS-CoV. It inhibited the viral entry into host cells by binding with the spike proteins and interfering with the SARS-CoV-23CL protease activity (124). Likewise, luteolin and quercetin could also interfere with the viral entry to its host cells (125). Furthermore, tetra-*O*-galloyl-β-d-glucose (TGG) and luteolin showed anti-SARS-CoV-2activities; TGG exhibited prominent anti-SARS-CoV-2 activity with an IC_50_ of 4.5 μM. These reports suggest that specific glycosylated flavonoids may play an effective role in inhibiting SARS-CoV-2replication activity. In summary, natural small molecules may be excellent opportunities for further optimization and potential clinical use against SARS-CoV, especially targeting 3CL protease. Additionally, traditional and alternative medicine may be explored for future drug development processes (126, 127). A recent review highlighted that a good number of medicinal plants and their herb-derived constituents have shown potential anti-SARS-CoV activity (non-clinical and pre-clinical study). Such agents are not only important to combat SARS-CoVs, but also play an important role in preventing viral attacks. However, there is a lack of adequate research on the development of anti-nCoV-19 agents from such natural products (128).

## Limitations

As there is a huge volume of therapeutic approaches for COVID-19, we may not cover all available therapeutic approaches. In addition, research results are dynamic and change as new evidence emerges. Second, only data based on the adult population and not the pediatric population were included in this review. Third, only articles/publications/translations from English were analyzed so some relevant international data might be missing.

## Conclusions

Coronavirus SARS-CoV-2 is, at present, the most important topic in medical research; from epidemiology to possible treatments, there are many unknowns in this pandemic. In terms of treatment, there are several potential molecules; from remdesivir, an antiviral previously tested for Ebola already approved by the FDA, to antiretrovirals used against HIV and antimalarials such as hydroxychloroquine. However, a large number of small studies, with different methodologies, cannot adequately identify the most effective and safe treatment.

Drug development, together with vaccine development and epidemiological research into viral infection, is an essential element in understanding and controlling SARS-COV-2. Interim results of clinical trials will be published in the coming months, and patients will be able to benefit from final results and approvals in the coming months, with the support of the relevant authorities. In addition to studying therapeutic molecules in this emerging infection, an important step in the management of SARS-COV-2 is the approval of possible treatments. In this regard, the agencies responsible for the evaluation and approval of US and EU medicines—FDA and EMA—have adopted measures to encourage a rapid, but effective, trial in the case of SARS-CoV-2 coronavirus, a situation that could have a beneficial impact on the evolution of the epidemic.

Worldwide, deaths among infected persons is increasing on a daily basis. Therefore, the current SARS-CoV-2 pandemic is related to a social together with its viral catastrophe. The control of the outbreaks of SARS-CoV-2 is now becoming a world challenge. The development of preventive and controlling remedies along with personal precautions are urgently needed to avoid SARS-CoV-2 infection.

## Author Contributions

MT, MN, IK, SM, MK-E-Z, TA, and MR contributed to drafting and writing the manuscript. MMu, EA, and MMa were responsible for the collection of relevant literatures. AD, EA, DC, and JS-R contributed to the conception of the figure, interpreted the results, and revised the manuscript critically for important intellectual content. MT, WC, DC, AD, and JS-R supervised the whole process. All authors contributed equally and agreed to the published version of the manuscript.

## Conflict of Interest

The authors declare that the research was conducted in the absence of any commercial or financial relationships that could be construed as a potential conflict of interest.

## Footnotes

^1^Coronavirus disease 2019 (COVID-19) treatment guideline.

## References

[1] Bogoch Ii Watts A Thomas-Bachli A Huber C Kraemer MUG Khan K. Pneumonia of unknown aetiology in Wuhan, China: potential for international spread via commercial air travel. J Travel Med. (2020) 27:taaa008. 10.1093/jtm/taaa008 31943059 PMC7107534

[2] Hui DS E IA Madani TA Ntoumi F Kock R Dar O. The continuing 2019-nCoV epidemic threat of novel coronaviruses to global health - the latest 2019 novel coronavirus outbreak in Wuhan, China. Int J Infect Dis. (2020) 91:264–66. 10.1016/j.ijid.2020.01.009 31953166 PMC7128332

[3] Chan JF Lau SK To KK Cheng VC Woo PC Yuen KY. Middle East respiratory syndrome coronavirus: another zoonotic betacoronavirus causing SARS-like disease. Clin Microbiol Rev. (2015) 28:465–522. 10.1128/CMR.00102-14 25810418 PMC4402954

[4] Elfiky AA Mahdy SM Elshemey WM. Quantitative structure-activity relationship and molecular docking revealed a potency of anti-hepatitis C virus drugs against human corona viruses. J Med Virol. (2017) 89:1040–7. 10.1002/jmv.24736 27864902 PMC7167072

[5] Docea AO Tsatsakis A Albulescu D Cristea O Zlatian O Vinceti M. A new threat from an old enemy: re-emergence of coronavirus. Int J Mol Med. (2020) 45:1631–43. 10.3892/ijmm.2020.4555 32236624 PMC7169834

[6] Goumenou M Sarigiannis D Tsatsakis A Anesti O Docea AO Petrakis D. COVID-19 in Northern Italy: an integrative overview of factors possibly influencing the sharp increase of the outbreak. Mol Med Rep. (2020) 22:20–32. 10.3892/mmr.2020.11079 32319647 PMC7248465

[7] World Health Organization Coronavirus Disease 2019 (COVID-19). Situation Report - 66. March 27, 2020. World Health Organization (WHO) Report (2020).

[8] Arsene AL Dumitrescu I-B Dragoi CM Udeanu DI Lupuliasa D Jinga V A new ERA for the therapeutic management of the ongoing COVID-19 pandemic. Farmacia. (2020) 68:185–96. 10.31925/farmacia.2020.2.1

[9] Tsatsakis A Petrakis D Nikolouzakis TK Docea AO Calina D Vinceti M. COVID-19, an opportunity to reevaluate the correlation between long-term effects of anthropogenic pollutants on viral epidemic/pandemic events and prevalence. Food Chem Toxicol. (2020) 136:111418. 10.1016/j.fct.2020.111418 32437891 PMC7211730

[10] Chinese Clinical Trials (2020). Available online at: http://www/chictr.org/enindex.aspx (accessed April 15, 2020).

[11] Reid CR Airo AM Hobman TC. The virus-host interplay: biogenesis of +RNA replication complexes. Viruses. (2015) 7:4385–413. 10.3390/v7082825 26287230 PMC4576186

[12] Farsalinos K Niaura R Le Houezec J Barbouni A Tsatsakis A Kouretas D. Nicotine and SARS-CoV-2: COVID-19 may be a disease of the nicotinic cholinergic system. Toxicol Rep. (2020) 7:658–63. 10.1016/j.toxrep.2020.04.012 32355638 PMC7192087

[13] Guo YR Cao QD Hong ZS Tan YY Chen SD Jin HJ. The origin, transmission and clinical therapies on coronavirus disease 2019 (COVID-19) outbreak - an update on the status. Mil Med Res. (2020) 7:11. 10.1186/s40779-020-00240-0 32169119 PMC7068984

[14] Meo SA Alhowikan AM Al-Khlaiwi T Meo IM Halepoto DM Iqbal M. Novel coronavirus 2019-nCoV: prevalence, biological and clinical characteristics comparison with SARS-CoV and MERS-CoV. Eur Rev Med Pharmacol Sci. (2020) 24:2012–9. 10.26355/eurrev_202002_20379 32141570

[15] Lam TT Shum MH Zhu HC Tong YG Ni XB Liao YS. Identifying SARS-CoV-2 related coronaviruses in Malayan pangolins. Nature. (2020). 10.1038/s41586-020-2169-0. [Epub ahead of print]. 32218527

[16] Zhang T Wu Q Zhang Z Probable pangolin origin of SARS-CoV-2 associated with the COVID-19 outbreak. Curr Biol. (2020) 30:1346–51.e2. 10.2139/ssrn.3542586 32197085 PMC7156161

[17] Lu H Stratton CW Tang YW. Outbreak of pneumonia of unknown etiology in Wuhan, China: the mystery and the miracle. J Med Virol. (2020) 92:401–2. 10.1002/jmv.25678 31950516 PMC7166628

[18] Zhang L Shen F-M Chen F Lin Z Origin and evolution of the 2019 novel coronavirus. Clin Infect Dis. (2020) ciaa112. 10.1093/cid/ciaa112 PMC7108176 32011673

[19] Read JM Bridgen JR Cummings DA Ho A Jewell CP Novel coronavirus 2019-nCoV: early estimation of epidemiological parameters and epidemic predictions. medRxiv. (2020). 10.1101/2020.01.23.20018549 PMC8165596 34053269

[20] Guan WJ Ni ZY Hu Y Liang WH Ou CQ He JX Clinical characteristics of coronavirus disease 2019 in China. N Engl J Med. (2020) 382:1708–20. 10.1056/NEJMoa2002032 32109013 PMC7092819

[21] Huang C Wang Y Li X Ren L Zhao J Hu Y. Clinical features of patients infected with 2019 novel coronavirus in Wuhan, China. Lancet. (2020) 395:497–506. 10.1016/S0140-6736(20)30183-5 31986264 PMC7159299

[22] Chen H Guo J Wang C Luo F Yu X Zhang W. Clinical characteristics and intrauterine vertical transmission potential of COVID-19 infection in nine pregnant women: a retrospective review of medical records. Lancet. (2020) 395:809–15. 10.1016/S0140-6736(20)30360-3 32151335 PMC7159281

[23] Han W Quan B Guo Y Zhang J Lu Y Feng G. The course of clinical diagnosis and treatment of a case infected with coronavirus disease 2019. J Med Virol. (2020) 92:461–3. 10.1002/jmv.25711 32073161 PMC7167012

[24] Docea AO Gofită E Calina D Zaharie S Valcea DI Mitru? P Autoimmune disorders due to double antiviral therapy with Peginterferon and Ribavirin in patients with hepatitis C virus infection. Farmacia J. (2016) 64:605–11.

[25] Corman VM Landt O Kaiser M Molenkamp R Meijer A Chu DKW Detection of 2019 novel coronavirus (2019-nCoV) by real-time RT-PCR. Euro Surveill. (2020) 25:2000045 10.2807/1560-7917.ES.2020.25.3.2000045 PMC6988269 31992387

[26] Liu J Liao X Qian S Yuan J Wang F Liu Y. Community transmission of severe acute respiratory syndrome coronavirus 2, Shenzhen, China, 2020. Emerg Infect Dis. (2020) 26:1320–3. 10.3201/eid2606.200239 32125269 PMC7258448

[27] Li Q Guan X Wu P Wang X Zhou L Tong Y. Early transmission dynamics in Wuhan, China, of novel coronavirus–infected pneumonia. N Engl J Med. (2020) 382:1199–207. 10.1056/NEJMoa2001316 31995857 PMC7121484

[28] Ashour HM Elkhatib WF Rahman MM Elshabrawy HA. Insights into the recent 2019 novel coronavirus (SARS-CoV-2) in light of past human coronavirus outbreaks. Pathogens. (2020) 9:186. 10.3390/pathogens9030186 32143502 PMC7157630

[29] Rodriguez-Palacios A Cominelli F Basson A Pizarro T Ilic S. Textile masks and surface covers-a'universal droplet reduction model'against respiratory pandemics. Front Med. (2020) 7:260. 10.1101/2020.04.07.20045617 32574342 PMC7267001

[30] Wu C Liu Y Yang Y Zhang P Zhong W Wang Y. Analysis of therapeutic targets for SARS-CoV-2 and discovery of potential drugs by computational methods. Acta Pharm Sin B. (2020) 10:766–88. 10.1016/j.apsb.2020.02.008 32292689 PMC7102550

[31] Skalny AV Rink L Ajsuvakova OP Aschner M Gritsenko VA Alekseenko SI. Zinc and respiratory tract infections: perspectives for COVID-19. Int J Mol Med. (2020) 46:17–26. 10.3892/ijmm.2020.4575 32319538 PMC7255455

[32] Huang M Tang T Pang P Li M Ma R Lu J Treating COVID-19 with chloroquine. J Mol Cell Biol. (2020) 12:322–5. 10.1093/jmcb/mjaa014 32236562 PMC7232130

[33] Chen J Liu D Liu L Liu P Xu Q Xia L. [A pilot study of hydroxychloroquine in treatment of patients with moderate COVID-19]. Zhejiang Da Xue Xue Bao Yi Xue Ban. (2020) 49:215–9. 32391667 10.3785/j.issn.1008-9292.2020.03.03 PMC8800713

[34] Uno Y. Camostat mesilate therapy for COVID-19. Int Emerg Med. (2020) 1–2. 10.1007/s11739-020-02345-9 32347443 PMC7188520

[35] Zhou Y Vedantham P Lu K Agudelo J Carrion R Jr Nunneley JW. Protease inhibitors targeting coronavirus and filovirusentry. Antiv Res. (2015) 116:76–84. 10.1016/j.antiviral.2015.01.011 25666761 PMC4774534

[36] Cao B Wang Y Wen D Liu W Wang J Fan G. A trial of lopinavir-ritonavir in adults hospitalized with severe covid-19. N Engl J Med. (2020) 382:1787–99. 10.1056/NEJMoa2001282 32187464 PMC7121492

[37] Lim J Jeon S Shin H-Y Kim MJ Seong YM Lee WJ Case of the index patient who caused tertiary transmission of coronavirus disease 2019 in Korea: the application of lopinavir/ritonavir for the treatment of COVID-19 pneumonia monitored by quantitative RT-PCR. J Korean Med Sci. (2020) 35:e88 10.3346/jkms.2020.35.e89 32080993

[38] Zhu Z Lu Z Xu T Chen C Yang G Zha T. Arbidol monotherapy is superior to lopinavir/ritonavir in treating COVID-19. J Infect. (2020) 81:e21–e23. 10.1016/j.jinf.2020.03.060 32283143 PMC7195393

[39] Lian N Xie H Lin S Huang J Zhao J Lin Q. Umifenovir treatment is not associated with improved outcomes in patients with coronavirus disease 2019: a retrospective study. Clin Microbiol Infect. (2020) 26:917–21. 10.1016/j.cmi.2020.04.026. [Epub ahead of print]. 32344167 PMC7182750

[40] Mehta P Mcauley D Brown M Sanchez E Tattersall R Manson J. Correspondence COVID-19: consider cytokine storm syndromes and. Lancet. (2020) 6736:19–20. 10.1016/S0140-6736(20)30628-0 32192578 PMC7270045

[41] Chen W A Randomized, Double-Blind, Placebo Parallel-Controlled Phase I/II Clinical Trial for Inactivated Novel Coronavirus Pneumonia vaccine (Vero cells). ChiCTR2000031809 (2020). Available online at: http://www.chictr.org.cn/

[42] Apeiron Biologics APEIRON's Respiratory Drug Product to Start Pilot Clinical Trial to Treat Coronavirus Disease COVID-19 in China. (2020). Available online at: https://www.apeiron-biologics.com

[43] Yao X Ye F Zhang M Cui C Huang B Niu P. *In vitro* antiviral activity and projection of optimized dosing design of hydroxychloroquine for the treatment of severe acute respiratory syndrome coronavirus 2 (SARS-CoV-2). Clin Infect Dis. (2020) ciaa237. 10.1093/cid/ciaa237. [Epub ahead of print]. PMC7108130 32150618

[44] Vincent MJ Bergeron E Benjannet S Erickson BR Rollin PE Ksiazek TG. Chloroquine is a potent inhibitor of SARS coronavirus infection and spread. Virol J. (2005) 2:69. 10.1186/1743-422X-2-69 16115318 PMC1232869

[45] Gordon CJ Tchesnokov EP Feng JY Porter DP Gotte M. The antiviral compound remdesivir potently inhibits RNA-dependent RNA polymerase from Middle East respiratory syndrome coronavirus. J Biol Chem. (2020) 295:4773–9. 10.1074/jbc.AC120.013056 32094225 PMC7152756

[46] Keyaerts E Li S Vijgen L Rysman E Verbeeck J Van Ranst M. Antiviral activity of chloroquine against human coronavirus OC43 infection in newborn mice. Antimicrob Agents Chemother. (2009) 53:3416–21. 10.1128/AAC.01509-08 19506054 PMC2715625

[47] Rogoveanu OC Calina D Cucu MG Burada F Docea AO Sosoi S. Association of cytokine gene polymorphisms with osteoarthritis susceptibility. Exp Ther Med. (2018) 16:2659–64. 10.3892/etm.2018.6477 30186498 PMC6122495

[48] Liu J Cao R Xu M Wang X Zhang H Hu H. Hydroxychloroquine, a less toxic derivative of chloroquine, is effective in inhibiting SARS-CoV-2 infection in vitro. Cell Discov. (2020) 6:16. 10.1038/s41421-020-0156-0 32194981 PMC7078228

[49] Yamamoto M Matsuyama S Li X Takeda M Kawaguchi Y Inoue JI. Identification of nafamostat as a potent inhibitor of middle east respiratory syndrome coronavirus S protein-mediated membrane fusion using the split-protein-based cell-cell fusion assay. Antimicrob Agents Chemother. (2016) 60:6532–9. 10.1128/AAC.01043-16 27550352 PMC5075056

[50] Burki T Outbreak of coronavirus disease 2019. Lancet Infect Dis. (2020) 20:292–3. 10.1016/S1473-3099(20)30076-1 32078809 PMC7128260

[51] Zhou Y Hou Y Shen J Huang Y Martin W Cheng F. Network-based drug repurposing for novel coronavirus 2019-nCoV/SARS-CoV-2. Cell Discov. (2020) 6:14–14. 10.1038/s41421-020-0153-3 32194980 PMC7073332

[52] NIH NIH Clinical Trial Shows Remdesivir Accelerates Recovery from Advanced COVID-19. National Institute of Allergy and Infectious Diseases (NIH) (2020). Available online at: https://www.niaid.nih.gov/news-events/ (accessed April 24, 2020).

[53] Gilead Gilead Announces Results From Phase 3 Trial of Investigational Antiviral Remdesivir in Patients With Severe COVID-19. (2020). Available online at: https://www.gilead.com/news-and-press/ (accessed April 20, 2020).

[54] Chu CM Cheng VC Hung IF Wong MM Chan KH Chan KS. Role of lopinavir/ritonavir in the treatment of SARS: initial virological and clinical findings. Thorax. (2004) 59:252–6. 10.1136/thorax.2003.012658 14985565 PMC1746980

[55] Yao TT Qian JD Zhu WY Wang Y Wang GQ. A systematic review of lopinavir therapy for SARS coronavirus and MERS coronavirus-A possible reference for coronavirus disease-19 treatment option. J Med Virol. (2020). 10.1002/jmv.25729 32104907 PMC7217143

[56] Blaising J Polyak SJ Pecheur EI. Arbidol as a broad-spectrum antiviral: an update. Antiviral Res. (2014) 107:84–94. 10.1016/j.antiviral.2014.04.006 24769245 PMC7113885

[57] Jieming QU Clinical Study of Arbidol Hydrochloride Tablets in the Treatment of Pneumonia Caused by Novel Coronavirus. NCT04260594 (2020). Available online at: https://clinicaltrials.gov/ (accessed April 1, 2020).

[58] Kadam RU Wilson IA. Structural basis of influenza virus fusion inhibition by the antiviral drug Arbidol. Proc Natl Acad Sci USA. (2017) 114:206–14. 10.1073/pnas.1617020114 28003465 PMC5240704

[59] Chen C Zhang Y Huang J Yin P Cheng Z Wu J Favipiravir versus Arbidol for COVID-19: a randomized clinical trial. medRxiv. (2020). 10.1101/2020.03.17.20037432

[60] Wang D Hu B Hu C Zhu F Liu X Zhang J. Clinical characteristics of 138 hospitalized patients with 2019 novel coronavirus-infected pneumonia in Wuhan, China. JAMA. (2020) 323:1061–9. 10.1001/jama.2020.1585 32031570 PMC7042881

[61] Xu X Han M Li T Sun W Wang D Fu B. Effective treatment of severe COVID-19 patients with tocilizumab. Proc Natl Acad Sci USA. (2020) 117:10970–5. 10.1073/pnas.2005615117 32350134 PMC7245089

[62] Rojas M Rodriguez Y Monsalve DM Acosta-Ampudia Y Camacho B Gallo JE. Convalescent plasma in covid-19: possible mechanisms of action. Autoimmun Rev. (2020) 19:102554. 10.1016/j.autrev.2020.102554 32380316 PMC7198427

[63] Tian X Li C Huang A Xia S Lu S Shi Z. Potent binding of 2019 novel coronavirus spike protein by a SARS coronavirus-specific human monoclonal antibody. Emerg Microb Infect. (2020) 9:382–5. 10.1080/22221751.2020.1729069 32065055 PMC7048180

[64] Bloch EM Shoham S Casadevall A Sachais BS Shaz B Winters JL. Deployment of convalescent plasma for the prevention and treatment of COVID-19. J Clin Investig. (2020) 130:2757–65. 10.1172/JCI138745 32254064 PMC7259988

[65] Arturo C Liise-Anne P. The convalescent sera option for containing COVID19. J Clin Invest. (2020) 130:1545–8. 10.1172/JCI138003 32167489 PMC7108922

[66] FDA Donate COVID-19 Plasma. U.S. Food and Drug Administration (FDA) (2020). Available online at: https://www.fda.gov/ (accessed March 24, 2020).

[67] NHS Who Can Donate Convalescent Plasma? NHS Blood and Transplant. (2020). Available online at: https://www.nhsbt.nhs.uk/ (accessed April 16, 2020).

[68] Arabi YM Alothman A Balkhy HH Al-Dawood A Aljohani S Al Harbi S. Treatment of Middle East Respiratory Syndrome with a combination of lopinavir-ritonavir and interferon-beta1b (MIRACLE trial): study protocol for a randomized controlled trial. Trials. (2018) 19:81. 10.1186/s13063-017-2427-0 29382391 PMC5791210

[69] Oslo University Hospital The Efficacy of Different Anti-viral Drugs in COVID 19 Infected Patients. NCT04321616 (2020). Available online at: https://clinicaltrials.gov/ (accessed March 24, 2020).

[70] Institut National De La Santé Et De La Recherche Médicale Trial of Treatments for COVID-19 in Hospitalized Adults (DisCoVeRy). NCT04315948 (2020). Available online at: https://clinicaltrials.gov/ (accessed March 11, 2020).

[71] Hoffmann-La Roche A Study to Evaluate the Safety and Efficacy of Tocilizumab in Patients With Severe COVID-19 Pneumonia (COVACTA). NCT04320615 (2020). Available online at: https://clinicaltrials.gov/ (accessed April 10, 2020).

[72] University of Aarhus The Impact of Camostat Mesilate on COVID-19 Infection (CamoCO-19). NCT04321096 (2020). Available online at: https://clinicaltrials.gov/ (accessed April 10, 2020).

[73] University Hospital Padova Efficacy of Nafamostat in Covid-19 Patients (RACONA Study) (RACONA). NCT04352400 (2020). Available online at: https://clinicaltrials.gov/ (accessed April 10, 2020).

[74] Health Commission of Guangdong Province for Chloroquine in the Treatment of Novel Coronavirus [Expert consensus on chloroquine phosphate for the treatment of novel coronavirus pneumonia]. Zhonghua Jie He He Hu Xi Za Zhi. (2020) 43:185–8. 10.3760/cma.j.issn.1001-0939.2020.03.009 32164085

[75] Mohammadi S Jafari B Asgharian P Martorell M Sharifi-Rad J. Medicinal plants used in the treatment of Malaria: a key emphasis to Artemisia, Cinchona, Cryptolepis, and Tabebuia genera. Phytother Res. (2020) 1–14. 10.1002/ptr.6628 32022345

[76] Stokkermans TJ Goyal A Bansal P Trichonas G. Chloroquine And Hydroxychloroquine Toxicity. In: StatPearls. Treasure Island: StatPearls Publishing (2020). 30725771

[77] McChesney EW. Animal toxicity and pharmacokinetics of hydroxychloroquine sulfate. Am J Med. (1983) 75:11–8. 10.1016/0002-9343(83)91265-2 6408923

[78] Gautret P Lagier J-C Parola P Hoang VT Meddeb L Mailhe M. Hydroxychloroquine and azithromycin as a treatment of COVID-19: results of an open-label non-randomized clinical trial. Int J Antimicrob Agents. (2020) 105949. 10.1016/j.ijantimicag.2020.105949. [Epub ahead of print]. 32205204 PMC7102549

[79] Ungureanu A Zlatian O Mitroi G Drocaş A Tîrcă T Călina D. Staphylococcus aureus colonisation in patients from a primary regional hospital. Mol Med Rep. (2017) 16:8771–80. 10.3892/mmr.2017.7746 29039613 PMC5779955

[80] Sanders JM Monogue ML Jodlowski TZ Cutrell JB. Pharmacologic treatments for coronavirus disease 2019 (COVID-19): a review. JAMA. (2020) 323:1824–36. 10.1001/jama.2020.6019 32282022

[81] Chen L Xiong J Bao L Shi Y. Convalescent plasma as a potential therapy for COVID-19. Lancet Infect Dis. (2020) 20:398–400. 10.1016/S1473-3099(20)30141-9 32113510 PMC7128218

[82] Beijing Chao Yang Hospital Efficacy and Safety of Corticosteroids in COVID-19. NCT04273321 (2020). Available online at: https://www.clinicaltrials.gov/ (accessed April 12, 2020).

[83] Stebbing J Phelan A Griffin I Tucker C Oechsle O Smith D. COVID-19: combining antiviral and anti-inflammatory treatments. Lancet Infect Dis. (2020) 20:400–2. 10.1016/S1473-3099(20)30132-8 32113509 PMC7158903

[84] Shetty AK. Mesenchymal stem cell infusion shows promise for combating coronavirus (COVID-19)- induced pneumonia. Aging Dis. (2020) 11:462–4. 10.14336/AD.2020.0301 32257554 PMC7069463

[85] Yang Y Islam MS Wang J Li Y Chen X. Traditional Chinese medicine in the treatment of patients infected with 2019-new coronavirus (SARS-CoV-2): a review and perspective. Int J Biol Sci. (2020) 16:1708–17. 10.7150/ijbs.45538 32226288 PMC7098036

[86] Boda D Docea AO Calina D Ilie MA Caruntu C Zurac S. Human papilloma virus: apprehending the link with carcinogenesis and unveiling new research avenues. Int J Oncol. (2018) 52:637–55. 10.3892/ijo.2018.4256 29393378 PMC5807043

[87] Gouglas D Thanh Le T Henderson K Kaloudis A Danielsen T Hammersland NC. Estimating the cost of vaccine development against epidemic infectious diseases: a cost minimisation study. Lancet Glob Health. (2018) 6:e1386–96. 10.1016/S2214-109X(18)30346-2 30342925 PMC7164811

[88] Gates B. The next epidemic — lessons from Ebola. N Engl J Med. (2015) 372:1381–4. 10.1056/NEJMp1502918 25853741

[89] Ratajczak W Niedzwiedzka-Rystwej P Tokarz-Deptuła B Deptuła W. Immunological memory cells. Central Eur J Immunol. (2018) 43:194–203. 10.5114/ceji.2018.77390 30135633 PMC6102609

[90] AminJafari A Ghasemi S. The possible of immunotherapy for COVID-19: a systematic review. Int Immunopharmacol. (2020) 83:106455. 10.1016/j.intimp.2020.106455 32272396 PMC7128194

[91] Calina D Docea AO Petrakis D Egorov AM Ishmukhametov AA Gabibov AG. Towards effective COVID-19 vaccines: updates, perspectives and challenges. Int J Mol Med. (2020) 46:3–16. 10.3892/ijmm.2020.4596 32377694 PMC7255458

[92] University of Oxford. A Study of a Candidate COVID-19 Vaccine (COV001). NCT04324606 (2020). Available online at: https://www.clinicaltrials.gov/ (accessed March 2, 2020).

[93] Du L He Y Zhou Y Liu S Zheng BJ Jiang S. The spike protein of SARS-CoV–a target for vaccine and therapeutic development. Nat Rev Microbiol. (2009) 7:226–36. 10.1038/nrmicro2090 19198616 PMC2750777

[94] Agnandji ST Huttner A Zinser ME Njuguna P Dahlke C Fernandes JF. Phase 1 trials of rVSV Ebola vaccine in Africa and Europe. N Engl J Med. (2016) 374:1647–60. 10.1056/NEJMoa1502924 25830326 PMC5490784

[95] Zhang Q Wang Y Qi C Shen L Li J Clinical trial analysis of 2019-nCoV therapy registered in China. J Med Virol. (2020) 92:540–5. 10.1002/jmv.25803 32108352 PMC7228274

[96] CanSino Biologics Inc Phase I Clinical Trial of a COVID-19 Vaccine in 18-60 Healthy Adults (CTCOVID-19). NCT04313127 (2020). Available online at: https://www.clinicaltrials.gov/ (accessed March 12, 2020).

[97] EPR JandJ Says Clinical Trials for COVID-19 Vaccine Candidate Will Begin by September. European Pharmaceutical Review (EPR) (2020). Available online at: https://www.europeanpharmaceuticalreview.com/ (acceseed March 12, 2020).

[98] BAT BAT Working on Potential COVID-19 Vaccine Through US Bio-Tech Subsidiary. A Better Tomorrow (BAT). (2020). Available online at: https://www.bat.com/ (accessed April 24, 2020).

[99] Euractiv British American Tobacco Working on COVID-19 Vaccine Using Tobacco Leaves. (2020). Available online at: https://www.euractiv.com/ (accessed April 3, 2020).

[100] Salehi B Capanoglu E Adrar N Catalkaya G Shaheen S Jaffer M. Cucurbits plants: a key emphasis to its pharmacological potential. Molecules. (2019) 24:1854. 10.3390/molecules24101854 31091784 PMC6572650

[101] Salehi B Sharifi-Rad J Capanoglu E Adrar N Catalkaya G Shaheen S Cucurbita plants: from farm to industry. Appl Sci. (2019) 9:3387 10.3390/app9163387

[102] Zhang MM Liu XM He L. Effect of integrated traditional Chinese and Western medicine on SARS: a review of clinical evidence. World J Gastroenterol. (2004) 10:3500–5. 10.3748/wjg.v10.i23.3500 15526373 PMC4576235

[103] Li SY Chen C Zhang HQ Guo HY Wang H Wang L. Identification of natural compounds with antiviral activities against SARS-associated coronavirus. Antiviral Res. (2005) 67:18–23. 10.1016/j.antiviral.2005.02.007 15885816 PMC7114104

[104] Vlad DC Popescu R Dumitrascu V Cimporescu A Vlad CS Vagvoelgyi C Phytocomponents identification in mistletoe (*Viscum album*.) young leaves and branches, by GC-MS and antiproliferative effect on HEPG2 and McF7 cell lines. Farmacia J. (2016) 64:82–7.

[105] Gitea D Vicas S Gitea MA Nemeth S Tit DM Pasca B HPLC screening of bioactives compounds and antioxidant capacity of different hypericum species. Revista de Chimie. (2018) 69:305–9. 10.37358/RC.18.2.6095

[106] Ma Q Huang W Zhao J Yang Z. Liu Shen Wan inhibits influenza a virus and excessive virus-induced inflammatory response via suppression of TLR4/NF-kappaB signaling pathway in vitro and in vivo. J Ethnopharmacol. (2020) 252:112584. 10.1016/j.jep.2020.112584 31972325

[107] Lin CW Tsai FJ Tsai CH Lai CC Wan L Ho TY. Anti-SARS coronavirus 3C-like protease effects of Isatis indigotica root and plant-derived phenolic compounds. Antiviral Res. (2005) 68:36–42. 10.1016/j.antiviral.2005.07.002 16115693 PMC7114321

[108] Chen CJ Michaelis M Hsu HK Tsai CC Yang KD Wu YC. Toona sinensis Roem tender leaf extract inhibits SARS coronavirus replication. J Ethnopharmacol. (2008) 120:108–11. 10.1016/j.jep.2008.07.048 18762235 PMC7127248

[109] Ryu YB Jeong HJ Kim JH Kim YM Park JY Kim D. Biflavonoids from Torreya nucifera displaying SARS-CoV 3CL(pro) inhibition. Bioorg Med Chem. (2010) 18:7940–7. 10.1016/j.bmc.2010.09.035 20934345 PMC7126309

[110] Cinatl J Morgenstern B Bauer G Chandra P Rabenau H Doerr HW. Glycyrrhizin, an active component of liquorice roots, and replication of SARS-associated coronavirus. Lancet. (2003) 361:2045–6. 10.1016/S0140-6736(03)13615-X 12814717 PMC7112442

[111] Wu CY Jan JT Ma SH Kuo CJ Juan HF Cheng YS. Small molecules targeting severe acute respiratory syndrome human coronavirus. Proc Natl Acad Sci USA. (2004) 101:10012–7. 10.1073/pnas.0403596101 15226499 PMC454157

[112] Hoever G Baltina L Michaelis M Kondratenko R Baltina L Tolstikov GA. Antiviral activity of glycyrrhizic acid derivatives against SARS-coronavirus. J Med Chem. (2005) 48:1256–9. 10.1021/jm0493008 15715493

[113] Amici C Di Caro A Ciucci A Chiappa L Castilletti C Martella V. Indomethacin has a potent antiviral activity against SARS coronavirus. Antivir Ther. (2006) 11:1021–30. 17302372

[114] Chen YH Chen AP Chen CT Wang AH Liang PH. Probing the conformational change of Escherichia coli undecaprenyl pyrophosphate synthase during catalysis using an inhibitor and tryptophan mutants. J Biol Chem. (2002) 277:7369–76. 10.1074/jbc.M110014200 11744728

[115] Kuo CJ Chi YH Hsu JT Liang PH. Characterization of SARS main protease and inhibitor assay using a fluorogenic substrate. Biochem Biophys Res Commun. (2004) 318:862–7. 10.1016/j.bbrc.2004.04.098 15147951 PMC7134607

[116] Wen CC Shyur LF Jan JT Liang PH Kuo CJ Arulselvan P. Traditional Chinese medicine herbal extracts of *Cibotium barometz, Gentiana scabra*, *Dioscorea batatas, Cassia tora*, and *Taxillus chinensis* inhibit SARS-CoV replication. J Tradit Complement Med. (2011) 1:41–50. 10.1016/S2225-4110(16)30055-4 24716104 PMC3942999

[117] Kakuda R Iijima T Yaoita Y Machida K Kikuchi M. Secoiridoid glycosides from *Gentiana scabra*. J Nat Prod. (2001) 64:1574–5. 10.1021/np010358o 11754617

[118] Kim JA Son NS Son JK Jahng Y Chang HW Jang TS. Two new secoiridoid glycosides from the rhizomes of *Gentiana scabra* Bunge. Arch Pharm Res. (2009) 32:863–7. 10.1007/s12272-009-1608-0 19557364

[119] Su PF Staniforth V Li CJ Wang CY Chiao MT Wang SY. Immunomodulatory effects of phytocompounds characterized by *in vivo* transgenic human GM-CSF promoter activity in skin tissues. J Biomed Sci. (2008) 15:813–22. 10.1007/s11373-008-9266-7 18622761

[120] Jin M Suh SJ Yang JH Lu Y Kim SJ Kwon S. Anti-inflammatory activity of bark of Dioscorea batatas DECNE through the inhibition of iNOS and COX-2 expressions in RAW264.7 cells via NF-kappaB and ERK1/2 inactivation. Food Chem Toxicol. (2010) 48:3073–9. 10.1016/j.fct.2010.07.048 20691245

[121] Su PF Li CJ Hsu CC Benson S Wang SY Aravindaram K. Dioscorea phytocompounds enhance murine splenocyte proliferation *ex vivo* and improve regeneration of bone marrow cells *in vivo*. Evid Based Complement Alternat Med. (2011) 2011:731308. 10.1093/ecam/neq032 21799689 PMC3137395

[122] Jin YH Cai L Cheng ZS Cheng H Deng T Fan YP. A rapid advice guideline for the diagnosis and treatment of 2019 novel coronavirus (2019-nCoV) infected pneumonia (standard version). Mil Med Res. (2020) 7:4. 10.1186/s40779-020-0233-6 32029004 PMC7003341

[123] Kim HY Eo EY Park H Kim YC Park S Shin HJ. Medicinal herbal extracts of Sophorae radix, Acanthopanacis cortex, Sanguisorbae radix and Torilis fructus inhibit coronavirus replication in vitro. Antivir Ther. (2010) 15:697–709. 10.3851/IMP1615 20710051

[124] Ho TY Wu SL Chen JC Li CC Hsiang CY. Emodin blocks the SARS coronavirus spike protein and angiotensin-converting enzyme 2 interaction. Antiviral Res. (2007) 74:92–101. 10.1016/j.antiviral.2006.04.014 16730806 PMC7114332

[125] Yi L Li Z Yuan K Qu X Chen J Wang G. Small molecules blocking the entry of severe acute respiratory syndrome coronavirus into host cells. J Virol. (2004) 78:11334–9. 10.1128/JVI.78.20.11334-11339.2004 15452254 PMC521800

[126] Ganjhu RK Mudgal PP Maity H Dowarha D Devadiga S Nag S. Herbal plants and plant preparations as remedial approach for viral diseases. Virusdisease. (2015) 26:225–36. 10.1007/s13337-015-0276-6 26645032 PMC4663710

[127] Denaro M Smeriglio A Barreca D De Francesco C Occhiuto C Milano G. Antiviral activity of plants and their isolated bioactive compounds: an update. Phytother Res. (2019) 34:742–68. 10.1002/ptr.6575 31858645

[128] Islam MT Sarkar C El-Kersh DM Jamaddar S Uddin SJ Shilpi JA. Natural products and their derivatives against coronavirus: a review of the non-clinical and pre-clinical data. Phytother Res. (2020). 10.1002/ptr.6700. [Epub ahead of print]. 32248575

